# Analysis and Correction of the Magnetometer’s Position Error in a Cross-Shaped Magnetic Tensor Gradiometer

**DOI:** 10.3390/s20051290

**Published:** 2020-02-27

**Authors:** Youyu Yan, Yan Ma, Jianguo Liu

**Affiliations:** School of Marine Science and Technology, Northwestern Polytechnical University, Xi’an 710072, China; yanyouyu@mail.nwpu.edu.cn (Y.Y.); liujianguo@nwpu.edu.cn (J.L.)

**Keywords:** magnetic dipole, magnetic gradient tensor, position error of magnetometers, locating error, error correction

## Abstract

When using the technique of magnetic gradient tensor measurements to obtain the position of magnetic objects, calibration of the magnetic tensor gradiometer plays a pivotal role in precisely locating the target, and extensive research has been carried out on this up to now. However, previous studies have always lacked sufficient discussion on the position error of magnetometers in magnetic tensor gradiometers caused by inaccurate installment of magnetometers. In this paper, we analyze and correct this position error based on a magnetic dipole source. The result of the simulation exemplifies that the magnetometer’s position error will affect the locating accuracy and, therefore, it is worth correcting this error. The relationship between position error and magnetic gradient tensor components is established, followed by an error correction method based on this relationship. Simulations illustrate that this method can effectively decrease the effect caused by the position error of magnetometers and improve the locating performance with locating error and magnetic moment errors dropping from 2 to 0.2 m and $6\times 10^{5}A\cdot m^{2}$ to $5\times 10^{4}A\cdot m^{2}$, respectively.

## 1. Introduction

Recently, the magnetic gradient tensor system has been extensively applied in geophysical exploration [1], such as underwater target detection and unexploded ordnance [2], due to the advantages of magnetic gradient tensor measurements, the unstoppable development of technology, and the improvement in various platforms [3]. However, the performance of the tensor system is adversely affected by the measurement error caused by specific factors, which are broadly divided into three aspects [4].

Firstly, an inherent error exists in the magnetic gradient tensor system in obtaining the magnetic gradient tensor components, as the magnetic gradient tensor is generally approximated by the numerical difference between two separated magnetometers instead of the differential. As a result, magnetic objects’ localization by magnetic field gradient tensor measurement always involves an inherent locating error. This locating error can be corrected by iterations based on the systematic position error distribution patterns [5]. Besides, the geometric configuration of the system will also affect the accuracy of the obtained data. Rong et al. used the hybrid model of an ellipsoid and magnetic dipole array to simulate and analyze the measurement performance of several typical magnetic gradient tensor systems with different geometric configurations, and proposed that the cross-shaped magnetic gradient tensor system has the characteristics of an optimal structure with minimum measurement error [6].

Secondly, systematic errors (scale factors, non-orthogonally, and bias error) of magnetometers due to the limited production process and processing level also cause the inaccuracy in measurements [7,8]. However, several theories on the mechanism of this error and its corresponding error calibration methods have been proposed and can thus be efficiently solved. In 2001, Gebre proposed a nonlinear, two-step estimation algorithm to calibrate a single solid-state magnetometer, making the magnetometer calibration achievable without rotating the loaded magnetometer platform [9]. Topaz et al. [10,11,12] analyzed the measurement errors caused by the magnetometer in detail and established the mathematical error model, basically fitting the real situation. Besides, calibration methods based on the functional link artificial neural network (FLANN) were also proposed. In 2019, YaXin et al. [13] proposed a novel calibration method based on tensor invariants in the nonuniform magnetic field without the extra device. The calibration parameters were estimated using the concept of the magnetic gradient tensor and corresponding rotational invariants combined with Levenberg–Marquardt optimization. As many effective and practical calibration methods for the magnetometer’s systematic error are present, we do not discuss these topics in this paper.

One of the important and not very well analyzed aspects of error is the inaccurate installment of magnetometers. Magnetometers may rotate slightly along with the installation center point, inevitably causing misalignment errors between magnetic sensors. Even a minor error can multiply serious errors in the measurement accuracy of the tensor system [14]. Mutual calibration between sensors is necessary before using obtained data for location and detection.

Researchers have proposed many fast and efficient calibration methods for misalignment errors until now. Chi et al. obtained the calibration matrix [15] by solving the orthogonal Procrustes problem; Qingzhu et al. proposed that calibration of misalignment errors can be completed just by three sets of measurement data with the same rotation period [16]. All these methods only consider the misalignment error when installing the sensor, but do not consider the position error of magnetometers caused by inaccurate installment. The position of the sensor’s center may not accurately be at its geometric center, or magnetometers may not be correctly installed at the required position, especially for the magnetic gradient tensor system with flexible baseline length in which magnetometers will be slightly inaccurately installed along its axis after each change in baseline. This error will lead to a difference between the actual baseline length and standard baseline length of the tensor system, which significantly affects the accuracy of the obtained magnetic gradient tensor and locating results.

Herein, we analyze the magnetometer’s position error in depth and simulate how this error influences the locating results. The mathematical relationship between the magnetic gradient tensor components and magnetometer’s error is derived. According to the established mathematical model of the magnetometer’s position error, the calibration method is proposed, and its feasibility is verified by simulation at the end of the paper.

The paper is structured as follows. First, the theory of the magnetic gradient tensor and location based on the magnetic gradient tensor is introduced. Afterward, the magnetometer’s position error and its influence on the location results are analyzed followed by a correction method for it. Finally, the simulation results of the above content are clearly shown.

## 2. Magnetic Gradient Tensor and Location Theory

When discussing the location of the magnetic target, the distance to the target is usually 3 times larger than the physical dimensions of the target, so the target can be regarded as a magnetic dipole source [17]. The magnitude of the magnetic field generated by a magnetic dipole is expressed as follows:

(1) $$B=\frac{\mu_{0}}{4\pi }(\frac{3\left(M•r\right)r}{r^{5}}-\frac{M}{r^{3}}).$$

where r=|r|, $\mu_{0}\approx 4\pi \times 10^{-7}\;H/m$, $M=(M_{x},M_{y},M_{z})$, and r represents the position vector. $\mu_{0}$ is the permeability of vacuum and M denotes the magnetic moment vector.

The magnetic gradient tensor G is defined as the vector gradient of the magnetic field vector B [3]. It is formally presented in Equation (2).

(2) $$G=\nabla B=\left[\begin{array}{ccc} G_{xx} & G_{xy} & G_{xz}\\ G_{yx} & G_{yy} & G_{yz}\\ G_{zx} & G_{zy} & G_{zz} \end{array}\right].$$

Equation (2) shows that the magnetic gradient tensor is symmetrical, and the sum of the three components of the principal diagonal is equal to 0, so only five of the nine components of the magnetic gradient tensor are independent [18], and it can be expressed by [19]

(3) $$G_{ij}=\frac{\mu_{0}}{4\pi }\left[-\frac{15(M•r)ij}{r^{7}}+\frac{3(M_{i}j+M_{j}i+(M•r)\delta_{ij})}{r^{5}}\right].$$

i,j represent x,y,z, and $\delta_{ij}=1$ for i=j and $\delta_{ij}=0$ for i≠j.

**Figure 1:**
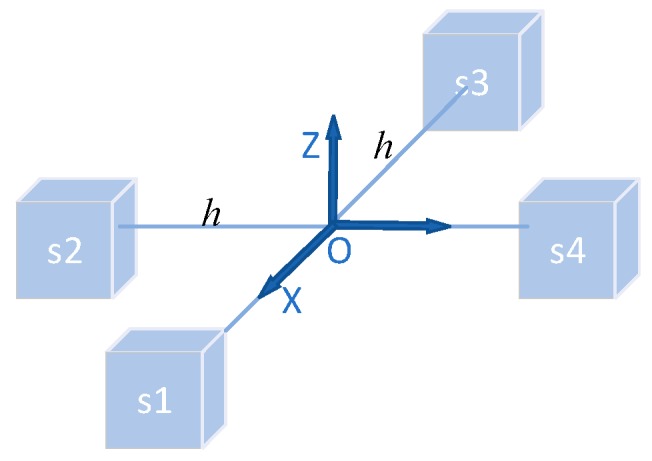
The schematic of the magnetic tensor gradiometer system.

The magnetic gradient tensor data can be measured by using a magnetic sensor array with a certain structure, such as a cross-shaped array, cube-shaped array, and three-dimensional cross-shaped array, to measure the magnitude of the magnetic field along the three orthogonal axes and calculate the difference in each direction. A schematic of the magnetic tensor gradiometer system is depicted in Figure 1. The formula of the cross-magnetic gradient tensor is shown as [20]

(4) $$G=\left[\begin{array}{ccc} \frac{B_{1x}-B_{3x}}{2h} & \frac{B_{1y}-B_{3y}}{2h} & \frac{B_{1z}-B_{3z}}{2h}\\ \frac{B_{2x}-B_{4x}}{2h} & \frac{B_{2y}-B_{4y}}{2h} & \frac{B_{2z}-B_{4z}}{2h}\\ \frac{B_{1z}-B_{3z}}{2h} & \frac{B_{2z}-B_{4z}}{2h} & -\frac{B_{1x}-B_{3x}}{2h}-\frac{B_{2y}-B_{4y}}{2h} \end{array}\right].$$

$B_{ij}(i=1,2,3,4;j=x,y,z)$ is the output of the magnetometer si, and h is the baseline length of the magnetic tensor gradiometer.

Euler deconvolution is a powerful technique for locating the target, which can be seen as a magnetic dipolar source, as we can directly estimate the target location from the raw data, and any initial estimation of parameters is not required in this method. The expression of the Euler deconvolution algorithm is [21]

(5) $$r=-3G^{-1}B.$$

We can locate the magnetic object exactly if we obtain the accurate magnetic gradient tensor components and the magnitude of the magnetic field of the target. The magnetic moment vector can also be estimated correspondingly once we obtain the location of the target [5].

(6) $$\left[\begin{array}{c} M_{x}\\ M_{y}\\ M_{z} \end{array}\right]=2\pi r\left[\begin{array}{ccc} 3x^{2}-2r^{2} & 3xy & 3xz\\ 3xy & 3y^{2}-2r^{2} & 3yz\\ 3xz & 3yz & 3z^{2}-2r^{2} \end{array}\right]\left[\begin{array}{c} B_{x}\\ B_{y}\\ B_{z} \end{array}\right].$$

## 3. The Analysis and Correction of Magnetometer’s Position Error

### 3.1. Analysis of Locating Error

In this paper, we analyze the influence of the magnetometer’s position error on localization accuracy based on the Euler deconvolution algorithm mentioned above.

**Figure 2:**
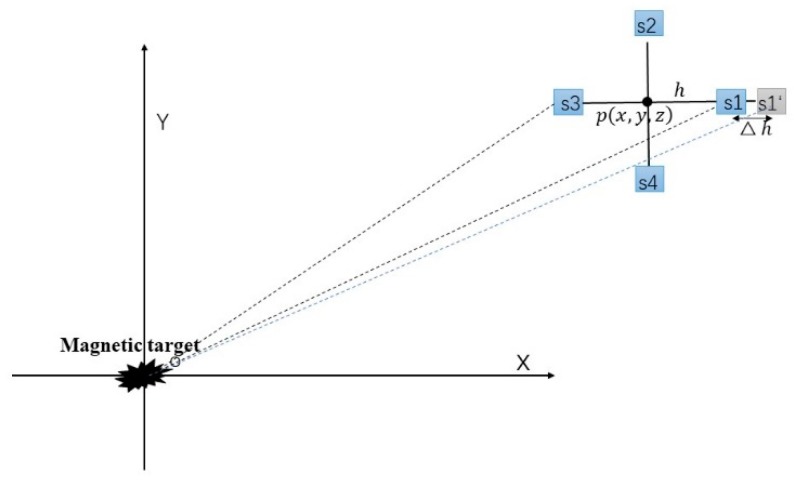
Overhead view of the experiment scene.

The magnetic gradient tensor measurement system constructed by four three-axis fluxgate sensors is located at the measuring point. Here, we assume that all the magnetometers are the standard sensors without any systematic errors, and there is no misalignment error among the magnetometer array. A target that can be modeled as a magnetic dipole is located at the origin of the coordinates. It is assumed that there exists a position error only in the magnetometer s1 when installing the sensor on the cross bracket (Figure 2). The accuracy of $G_{xx},G_{xy},G_{xz}$ obtained by this system suffers. According to Equations (1) and (4), these three tensor components, which are acquired by this magnetic tensor gradiometer with the position error of the sensor s1, can be expressed as follows:

(7) $$\left[\begin{array}{c} G_{xx}{}^{\prime }\\ G_{xy}{}^{\prime }\\ G_{xz}{}^{\prime } \end{array}\right]=\frac{1}{2h}\frac{\mu_{0}}{4\pi r^{7}}\left[\begin{array}{ccc} (-15x^{2}+9r^{2})Q & 3yr^{2}(2h+\Delta h)-15yxQ & 3zr^{2}(2h+\Delta h)-15zxQ\\ 3yr^{2}(2h+\Delta h)-15xyQ & (-15y^{2}+3r^{2})Q & -15zyQ\\ 3zr^{2}(2h+\Delta h)-15xzQ & -15yzQ & (-15z^{2}+3r^{2})Q \end{array}\right]\left[\begin{array}{l} M_{x}\\ M_{y}\\ M_{z} \end{array}\right].$$

where Q=2hx+hΔh+xΔh. $G_{xx}{}^{\prime }$, $G_{xy}{}^{\prime }$ and $G_{xz}{}^{\prime }$ denote incorrect tensor components. Δh represents the position error of the magnetometer s1. The specific derivation of Equation (7) is given in Appendix A.

The position error of the magnetometer s1 causes errors in the measured magnetic gradient tensor components, which lead to the inaccuracy in the locating results. Equation (5) can be written in a scalar form and, according to the total differential theory, location errors of the target along the three axes can be written as follows:

(8) $$\Delta x=\frac{\partial x}{\partial G_{xx}}\Delta G_{xx}+\frac{\partial x}{\partial G_{xy}}\Delta G_{xy}+\frac{\partial x}{\partial G_{xz}}\Delta G_{xz}\;.$$

(9) $$\Delta y=\frac{\partial y}{\partial G_{xx}}\Delta G_{xx}+\frac{\partial y}{\partial G_{xy}}\Delta G_{xy}+\frac{\partial y}{\partial G_{xz}}\Delta G_{xz}\;.$$

(10) $$\Delta z=\frac{\partial z}{\partial G_{xx}}\Delta G_{xx}+\frac{\partial z}{\partial G_{xy}}\Delta G_{xy}+\frac{\partial z}{\partial G_{xz}}\Delta G_{xz}.$$

According to Equation (7), we obtain the expression of $\Delta G_{xi}$ as

(11) $$\Delta G_{xi}=G_{xi}-G_{xi}{}^{\prime }\approx \frac{G_{xi}}{2h}\Delta h+\frac{\mu_{0}}{8\pi }(\frac{3M_{x}+3\delta_{ix}M_{x}}{r^{5}}-\frac{15\left(M_{x}x+M_{y}y+M_{z}z\right)i}{r^{7}})\Delta h.$$

It is known from Equation (11) that the magnitude of the magnetic gradient tensor error is mainly determined by the first part $\frac{G_{xi}}{2h}\Delta h$, and $\Delta G_{xi}$ will be larger when the magnetic gradient tensor components are larger or the baseline distance is smaller.

### 3.2. The Correction Method for Magnetometer’s Position Error

In summary, the relationship between the measured tensor data with the magnetometer’s position error is established as $G_{ij}{}^{\prime }=f(x,y,z,M_{x},M_{y},M_{z},h,\Delta h)$.

We can rotate the magnetic tensor gradiometer around the Z-axis to obtain M (M≥2) groups of measured magnetic gradient tensor data:

(12) $$N=\left[\begin{array}{cccccc} N_{xx\theta 1} & N_{xx\theta 2} & N_{xx\theta 3} & \cdots & N_{xx\theta_{\left(M-1\right)}} & N_{xx\theta_{M}}\\ N_{xy\theta 1} & N_{xy\theta 2} & N_{xy\theta 3} & \cdots & N_{xy\theta_{\left(M-1\right)}} & N_{xy\theta_{M}}\\ N_{xz\theta 1} & N_{xz\theta 2} & N_{xz\theta 3} & \cdots & N_{xz\theta_{\left(M-1\right)}} & N_{xz\theta_{M}} \end{array}\right].$$

The theoretical inaccurate magnetic gradient tensor data after rotation can be calculated according to Equation (7):

Then, we can establish the error matrix between the actual measured tensor data and the calculated ones:

(13) $$E=\left[\begin{array}{ccc} N_{xx\theta 1}-G_{xx\theta 1}{}^{\prime } & N_{xx\theta 2}-G_{xx\theta 2}{}^{\prime } & \cdots \\ N_{xy\theta 1}-G_{xy\theta 1}{}^{\prime } & N_{xy\theta 2}-G_{xy\theta 2}{}^{\prime } & \cdots \\ N_{xz\theta 1}-G_{xz\theta 1}{}^{\prime } & N_{xz\theta 3}-G_{xy\theta 3}{}^{\prime } & \cdots \end{array}\right].$$

Finally, the error coefficient Δh can be obtained by solving this nonlinear Equation (14) with a suitable method:

(14) $$F=\min\{E^{T}•E\}.$$

## 4. Numerical Simulation

### 4.1. Influence Assessment

In order to investigate the influence of the magnetometers’ position error on locating, the simulation experiment is conducted as follows.

Simulation setup:

- A magnetic object, which can be seen as a magnetic dipole, is used as a target and placed at the origin of the coordinates. Its magnetic moment is assumed as $M_{x}=4.5\times 10^{7}\;A\cdot m^{2}$, $M_{y}=1\times 10^{5}\;A\cdot m^{2}$, and $M_{z}=1\times 10^{6}\;A\cdot m^{2}$.
- The baseline length of the magnetic gradient tensor system is 0.5 m and the precision of the magnetometer is 1 pT.
- A 600 m straight survey line containing 100 observation points is designed. It starts from point (−300,20,100) m to point (300,20,100) m.

**Figure 3:**
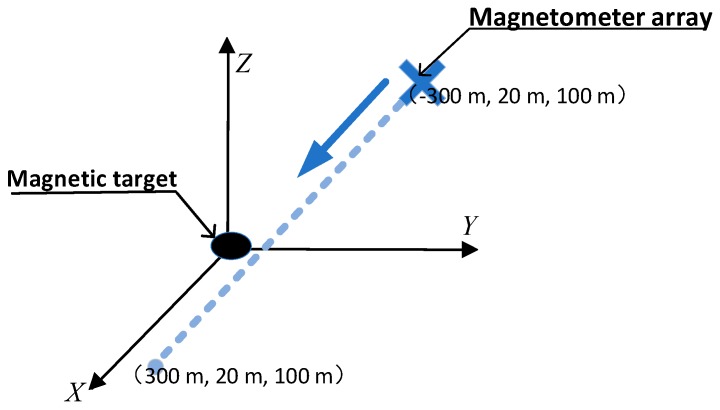
The schematic of the simulation experiment.

A schematic of the simulation experiment is shown in Figure 3.

The values of the magnetic gradient tensor and magnetic field intensity formed by the magnetic target in each observation point are calculated respectively through Equations (4) and (1). The location information of the target in each observation point can be estimated through Equation (5). Then, the magnetic moment of the target can also be obtained by Equation (6). In order to assess the locating performance, the location error and magnetic moment error of the target are defined as

(15) $$err_{location}=r_{true}-r_{estimated}.$$

(16) $$err_{magnetic\;moment}=M_{true}-M_{estimated}.$$

**Figure 4:**
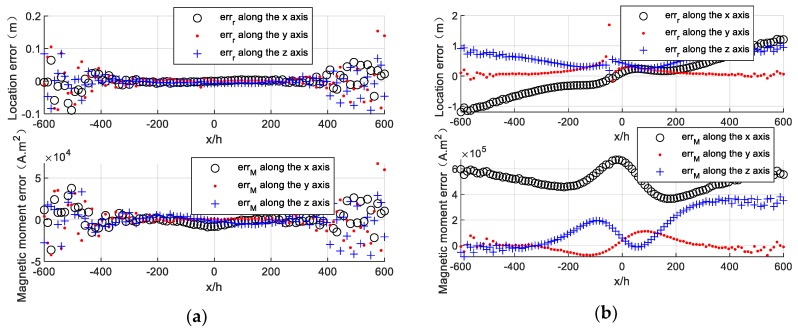
Locating results by using Euler deconvolution. (**a**) Locating without position error. (**b**) Locating with position error.

The location error and magnetic moment error of the target along the survey line are calculated and are shown in Figure 4a. The target locating based on Euler deconvolution has a high accuracy with low location error (less than 0.2 m) and comparatively low magnetic moment error (less than $5\times 10^{4}\;A\cdot m^{2}$), which can be seen in Figure 4a.

The position error of the magnetometer s1 (Δh=0.005 m, Δh/2h=0.5%) is added in the magnetic tensor gradiometer and the simulation experiment is carried out again. The simulation result is shown in Figure 4b. The locating accuracy seriously degrades compared to Figure 4a. The location error and magnetic moment error of the target increase to 2 m and $6\times 10^{5}\;A\cdot m^{2}$, respectively, due to the presence of the magnetometer’s position error.

**Figure 5:**
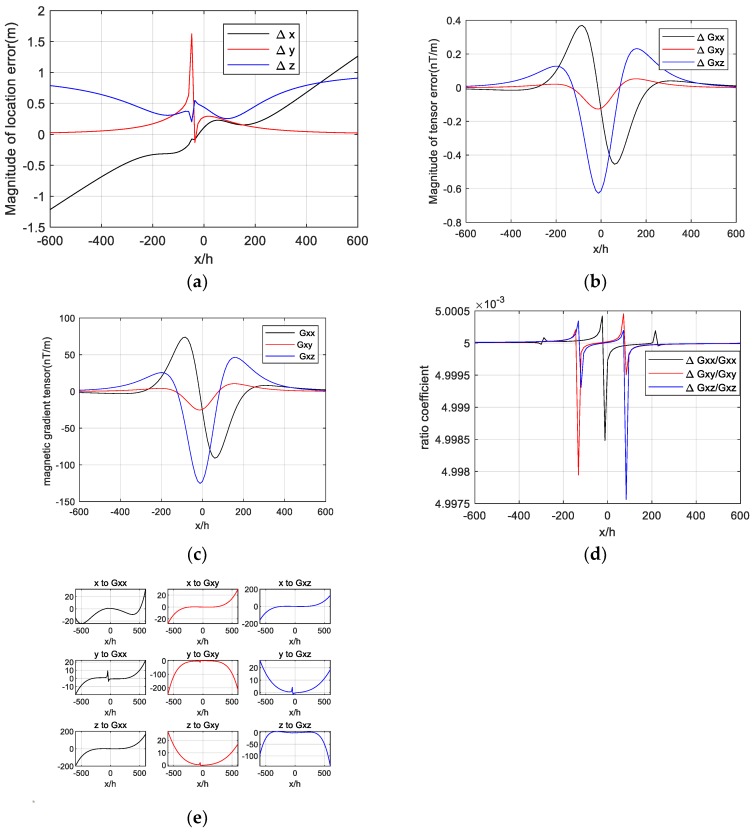
Influence analysis. (**a**) The magnitude of location error, (**b**) the magnitude of tensor error, (**c**) the magnetic gradient tensor, (**d**) ratio coefficient between gradient tensor error and gradient tensor, and (**e**) differential of position vector to magnetic gradient tensor.

We can calculate the location error of the target caused by the position error of the magnetometer s1 in each observation point through Equations (8), (9), and (10). The result is shown in Figure 5a, which is nearly the same as the experimental results in Figure 4b. The magnetic gradient tensor and magnitude of the gradient tensor error along the survey line are calculated through Equation (4) and Equation (11) and are shown in Figure 5b,c. The differentiation of the target’s position vector to the magnetic gradient tensor is drawn and shown in Figure 5e.

The tendency of the gradient tensor error is the same as that of the magnetic gradient tensor according to Figure 5b,c, and the ratio coefficient between them is almost equal to Δh/2h=0.5%, which can be seen in Figure 5d. This means that the magnitude of the magnetic gradient tensor error caused by the magnetometer’s position error is mainly determined by the first part $\frac{G_{xi}}{2h}\Delta h$ in Equation (11). Thus, the formula of $\Delta G_{xi}$ can be simplified as follows:

(17) $$\Delta G_{xi}=G_{xi}-G_{xi}{}^{\prime }\approx \frac{G_{xi}}{2h}\Delta h.$$

As shown in Figure 5e, we can see the tendency of the differential variation of location vectors x, y, and z to $G_{xx}$, $G_{xy}$ and $G_{xz}$ respectively. Their values are almost zero in the near-target area, while they sharply surge in the far target area. The location errors of the target in Figure 5a is determined by the differential of location vectors to the magnetic gradient tensor and the magnetic gradient tensor error.

### 4.2. Error Correction

In this section, a simulation experiment is designed to test the proposed correction method.

Simulation setup:

- A magnetic object with known magnetic moment ($M_{x}=4.5\times 10^{7}A\cdot m^{2}$, $M_{y}=1\times 10^{5}\;A\cdot m^{2}$, and $M_{z}=1\times 10^{6}\;A\cdot m^{2}$) is placed at the origin of the coordinates.
- Preset Δh=0.001m, Δh=0.003m, ……, Δh=0.01m.
- The baseline length of the magnetometer array is 0.5 m and the precision of the magnetometer is 1 pT. The measurement points, which are used to estimate Δh, can be selected arbitrarily. Herein, two points, (20, 20, 100) and (−300, 20, 100) m, with the same Y and Z of the survey line in Section 4.1, are selected to exemplify the correction method.

The magnetometer array is rotated every 30° around the Z-axis to obtain more than 6 sets of measured magnetic gradient tensor values. The unconstrained nonlinear equation is established according to Equation (14) and the error parameters are estimated. The solution error of Δh is defined as

(18) $$err_{\Delta h}=\left|\Delta h_{preset}-\Delta h_{estimated}\right|.$$

The simulation results are listed in Table 1.

As shown in Table 1, the estimated error parameters are basically equal to the theoretical data at these two points. This means that Δh can be successfully obtained at both these points and the calibration method proposed in Section 3.2 is feasible. The solution errors of Δh at the point (−300, 20, 100) m are much smaller, compared to the results at point (20, 20, 100) m, presumably because the distance between the point (−300, 20, 100) m and the magnetic dipole is larger. Equation (7) is deducted based on the assumption that the baseline length of the magnetometer array reaches comparatively close to zero. When the distance between the measurement point and the magnetic dipole is large enough, the baseline length of the magnetometer array can be seen as infinitesimal, and the inaccurate magnetic tensor data calculated by Equation (7) can better fit the real measured data. Therefore, the estimation of Δh has a better performance.

**Figure 6:**
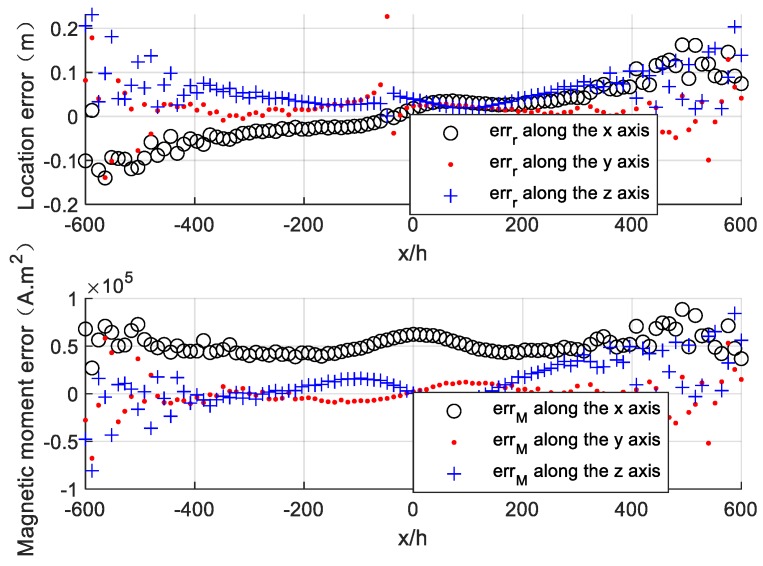
Locating results after calibration.

We use the estimated result of point (20, 20, 100) m to correct the magnetic gradient tensor along the survey line in Section 4.1 according to Equation (17) and use corrected data to carry out the locating experiment again. The locating result after correction is presented in Figure 6. The location errors and the magnetic moment errors of the target both largely declined after error correction.

## 5. Conclusions

The position error of magnetometers in the magnetic gradient tensor system was analyzed based on the model of a magnetic dipole. It can be seen from the simulation results that this error will seriously affect the locating accuracy, so it is highly necessary to analyze and correct such an error for precision. The function between position error and magnetic gradient tensor components is established. An error calibration method based on this function is proposed and this method is profound for further improving the capacity of the tensor system and performance of the localization algorithm.

## Figures and Tables

**Table 1:** The preset and estimated parameters in the simulation.

Number	Preset Δh(m)	(20, 20, 100) m		(−300, 20, 100) m	
		Estimated Δh(m)	$err_{\Delta h}(m)$	Estimated Δh(m)	$err_{\Delta h}(m)$
1	0.001	0.0007	0.0003	0.001	0
2	0.002	0.0017	0.0003	0.0019	0.0001
3	0.003	0.0027	0.0003	0.0029	0.0001
4	0.004	0.0036	0.0004	0.0039	0.0001
5	0.005	0.0045	0.0005	0.0049	0.0001
6	0.007	0.0062	0.0008	0.0068	0.0002
7	0.008	0.0071	0.0009	0.0077	0.0003
8	0.01	0.0089	0.0011	0.0096	0.0004

**Figure A1:**
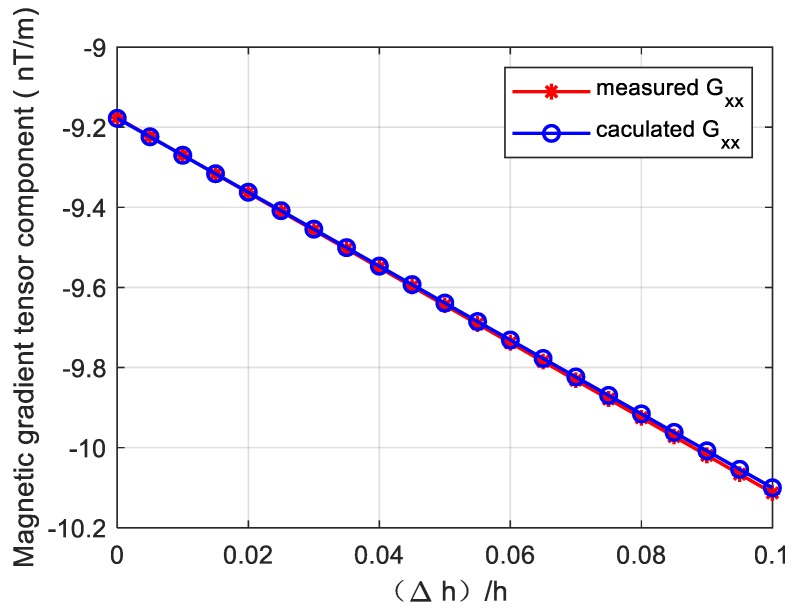
The simulation results of fitting degree.

## Appendix A

Derivation of Equation (7):

(A1) $$\begin{array}{ll} B_{1x}{}^{\prime } & =\frac{\mu_{0}}{4\pi }\left(\frac{3\left(M_{x}(x+h+\Delta h)+M_{y}y+M_{z}z\right)(x+h+\Delta h)-M_{x}{\left(x+h+\Delta h\right)}^{2}-M_{x}y^{2}+M_{x}z^{2}}{{\left({\left(x+h+\Delta h\right)}^{2}+y^{2}+z^{2}\right)}^{\frac{5}{2}}}\right)\\ & =\frac{\mu_{0}}{4\pi }(f(x+h+\Delta h)g(x+h+\Delta h)) \end{array}$$

(A2) $$\begin{array}{ll} B_{3x} & =\frac{\mu_{0}}{4\pi }\left(\frac{3\left(M_{x}(x-h)+M_{y}y+M_{z}z\right)(x-h)-M_{x}{\left(x-h\right)}^{2}-M_{x}y^{2}+M_{x}z^{2}}{{\left({\left(x-h\right)}^{2}+y^{2}+z^{2}\right)}^{\frac{5}{2}}}\right)\\ & =\frac{\mu_{0}}{4\pi }(f(x-h)g(x-h)) \end{array}$$

$B_{1x}{}^{\prime }$ and $B_{3x}$ denote the measured magnetic field intensity by senor s1, which has the position error, and sensor s3, respectively. We can write the mathematical expression of $B_{1x}{}^{\prime }$ and $B_{3x}$ according to Equation (1), and $G_{xx}{}^{\prime }$ can be written as Equation (A3) according to Equation (4).

(A3) $$\begin{array}{ll} G_{xx}{}^{\prime } & =\underset{2h\to 0}{\lim}\frac{B_{1x}{}^{\prime }-B_{3x}}{2h}=\frac{\mu_{0}}{4\pi }\underset{2h\to 0}{\lim}\frac{\left(f(x+h+\Delta h)g(x+h+\Delta h)-f(x-h)g(x-h)\right)}{2h}\\ & =\frac{\mu_{0}}{4\pi }\underset{2h\to 0}{\lim}\frac{1}{2h}(f(x+h+\Delta h)g(x+h+\Delta h)-f(x-h)g(x+h+\Delta h)\\ & \;-f(x-h)g(x-h)+f(x-h)g(x+h+\Delta h)) \end{array}$$

Simplify Equation (A3):

(A4) $$\begin{array}{ll} G_{xx}{}^{\prime }=\frac{\mu_{0}}{4\pi }\underset{2h\to 0}{\lim}\frac{1}{2h} & \{(2M_{x}(4xh+2h\Delta h+\Delta h^{2}+2x\Delta h)+3M_{y}y(2h+\Delta h)+3M_{z}z(2h+\Delta h))r^{-5}\\ & -\frac{5}{2}A•(4hx+\Delta h^{2}+2h\Delta h+2x\Delta h)r^{-7}\\ & -A•\frac{35}{8}({\left(2hx+h^{2}+\Delta h^{2}+2h\Delta h+2x\Delta h\right)}^{2}-(-2hx+h^{2}))r^{-9}+\cdots \} \end{array}$$

where $A=3\left(M_{x}x+M_{y}y+M_{z}z\right)x-M_{x}x^{2}-M_{x}y^{2}+M_{x}z^{2}$.

When 2h reaches extremely close to zero, Equation (A4) can be simplified as

(A5) $$\begin{array}{l} G_{xx}{}^{\prime }\approx \frac{\mu_{0}}{4\pi }\frac{1}{2h}\{(2M_{x}(4xh+2h\Delta h+2x\Delta h)+3M_{y}y(2h+\Delta h)+3M_{z}z(2h+\Delta h))r^{-5}\\ +(3(M_{x}x+M_{y}y+M_{z}z)x-M_{x}x^{2}-M_{x}y^{2}-M_{x}z^{2})(-\frac{5}{2}(4hx+2h\Delta h+2x\Delta h)r^{-7})\} \end{array}.$$

The mathematical expression of $G_{xy}{}^{\prime }$ and $G_{xz}{}^{\prime }$ can be obtained in the same way. In order to test the fitting degree of measured data and data calculated by the mathematical method, the simulation is carried out and the result is shown in Figure A1. The calculated data fit the measured data well when Δh is small relative to h according to the simulation result.

The simulation conditions are listed as follows:

- The magnetic moment of the magnetic object: $M_{x}=4.5\times 10^{7}A\cdot m^{2}$, $M_{y}=1\times 10^{5}\;A\cdot m^{2}$, and $M_{z}=1\times 10^{6}\;A\cdot m^{2}$.
- h=0.2m and $0\leq \frac{\Delta h}{h}\leq 0.1$.
- The observation point is arbitrarily selected as (20,10,10)m.
